# Hand-assisted bilateral nephrectomy in a patient with adult polycystic kidney disease

**DOI:** 10.1590/S1516-31802002000600007

**Published:** 2002-11-01

**Authors:** Marcello Alves Pinto, Roberto Vaz Juliano, Marcos Tobias-Machado, Milton Borrelli, Eric Roger Wroclawski.

**Keywords:** Nephrectomy, Laparoscopy, Polycystic Kidney Disease, Nefrectomia, Laparoscopia, Doença, Renal, Policística. Adulto

## Abstract

**CONTEXT:**

Dominantly autosomal polycystic disease is characterized by multiple bilateral and nonfunctional cysts, which lead to progressive kidney failure.

**OBJECTIVE:**

Our objective was to report on a case of hand-assisted bilateral nephrectomy in a 28-year-old female patient with adult polycystic disease and recurring pyelonephritis in a kidney transplant program.

**CASE REPORT:**

A hand-assisted bilateral nephrectomy was performed through a supra-umbilical median incision of approximately 6 cm, and with 3 ports of 10 mm. The length of the surgery was 3 hours and 15 minutes. The kidneys were removed after the aspiration of some cysts through the supra-umbilical incision. Pain control was achieved via the use of analgesics. The blood loss during surgery was 160 ml. During the postoperative period, the patient developed right-side pneumothorax, which was drained with no further occurrence. This drain was kept in place for 48 hours. The length of hospitalization was 4 days.

## RESUMO

**CONTEXTO:**

A doença policística autossômica dominante é caracterizada por cistos múltiplos bilaterais e não-funcionantes, que levam a uma perda progressiva da função renal.

**TIPO DE ESTUDO:**

Relato de caso.

**OBJETIVO:**

Relatar um caso de nefrectomia bilateral assistida com a mão em paciente do sexo feminino de 28 anos e com quadro de pielonefrite de repetição em programa de transplante renal.

**RELATO DE CASO:**

Foi realizado nefrectomia bilateral assistida com a mão em paciente com doença policística renal do adulto. A cirurgia foi realizada através de incisão mediana de aproximadamente 6 cm supra-umbilical (acesso previamente planejado no pré-operatório considerando acesso externo ao hilo no caso de necessidade devido ao grande volume dos rins) e três portais de 10 mm. O tempo cirúrgico foi de três horas e 15 minutos, sendo que o rim foi dissecado pela técnica assistida com a mão bilateralmente. As unidades renais foram retiradas após a aspiração de alguns cistos pela inci-são supra-umbilical. O controle da dor foi feito por meio de analgésicos leves, o tempo de internação hospitalar foi de quatro dias. O sangramento médio foi de 160 ml, e como intercorrência no pós-operatório, a paciente evoluiu com pneumotórax à direita, drenado sem intercorrências. O dreno perma-neceu por 48 horas.

## INTRODUCTION

The use of minimally invasive techniques in urology has become a reality since the 1990s. Patients with adult polycystic disease and symptoms of pain or discomfort can benefit from these minimally invasive procedures through decortication surgery, performed on kidney cysts via laparoscopy with good results regarding pain relief.^1^

There are some situations in which nephrectomy is necessary in patients with kidney failure in its final stage, especially in cases of hard-to-control severe hypertension, recurring pyelonephritis, severe protein loss,^1,2,3^ and enlarged kidney size causing respiratory or gastric discomfort.^3^

Bilateral nephrectomy by conventional access is normally performed via bilateral lumbotomy, as well as through a median xipho- pubic incision, with a high morbidity rate.^3^

Our objective was to report on a case of hand-assisted bilateral laparoscopic nephrectomy, describing the technique and commenting on this surgical option.

## CASE REPORT

**Figure 1:**
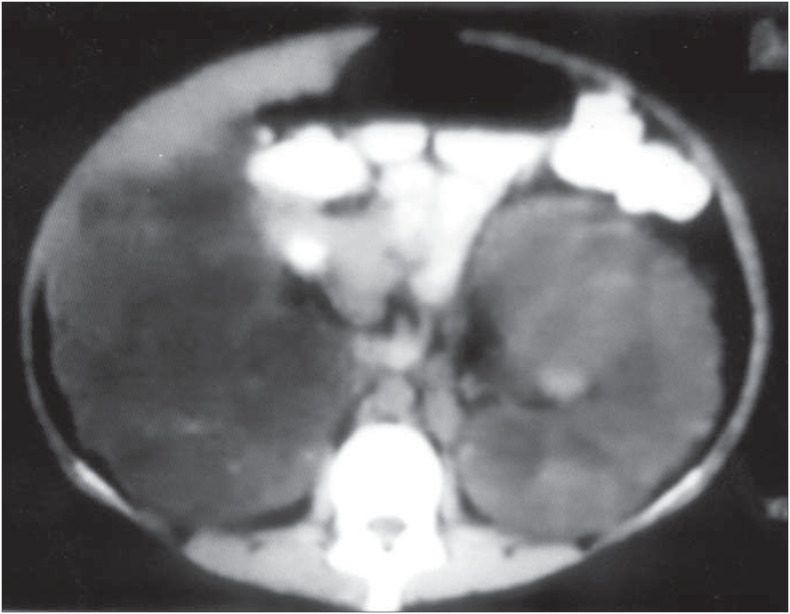
Appearance of the kidneys via computed tomography scan.

The patient was a 28-year-old female, with diagnosed adult polycystic disease, at the final stage of kidney failure and undergoing dialysis. This patient was in a kidney transplant program when she presented two episodes of pyelonephritis within 3 months. She was treated with antibiotics (quinolone, Cipro^®^), which led to complete remission of the condition. At the physical examination she presented palpable kidneys at both sides, in the iliac fossae. When preoperative examinations were performed, an abdominal computed tomography scan revealed very enlarged kidneys (26 cm on the right, 28 cm on the left), which had moved the colons bilaterally (Figure 1). Cistography of the ureter did not reveal any vesicoureteral reflux.

**Figure 2:**
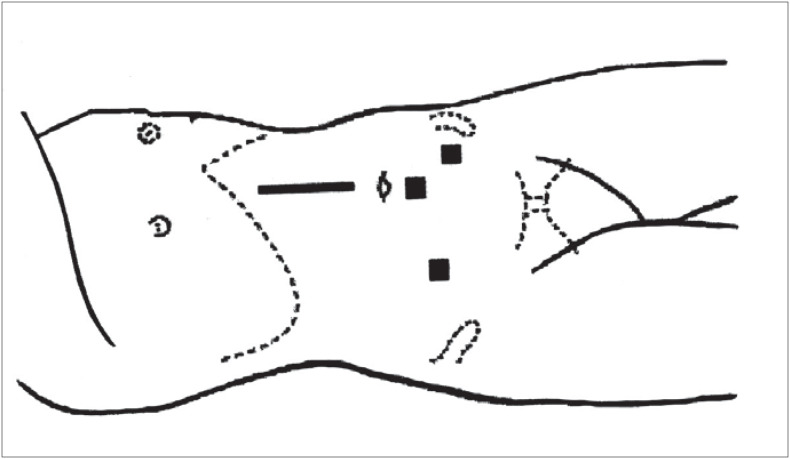
Ports that provided bilateral access to the kidneys.

## TECHNIQUE

The patient was placed in horizontal dorsal decubitus with hyperextension of the epigastric region by placing a cushion in the area of the twelfth rib.

The surgical strategy used involved the making of a supra-umbilical incision of approximately 6 cm, in order to insert the hand. This location was chosen for the incision as a result of pre-surgery planning done using the computed tomography scan, so as to deal with possible surgical difficulties that might arise because of the large volume of the kidneys. In addition to this incision, three other laparoscopic ports were opened: one of 10 mm in the infra-umbilical region, for the insertion of the optics, and another 2 ports of 5 mm to the left and 10 mm to the right, located midway between the iliac crest and the umbilical scar.

The surgeon used his left hand to dissect the right kidney, and his right hand to dissect the left kidney. The colons were bilaterally mobilized and, by means of dissection using laparoscopic scissors, the kidney was totally isolated using efficient, safe, and speedy hand action.

The ureters were isolated and sectioned bilaterally, and the renal hilum was reached via the open-surgery route through supra-umbili-cal incision. This was done because the large volume of the kidneys prevented safe access by laparoscopy. This maneuver was accomplished through the placement of manual retractor ligatures for the vessels, under direct vision.

The kidney was removed via the hand access port after the sectioning and external aspiration of a few kidney cysts, with no leakage of liquid to the peritoneal cavity.

The length of surgery was 3 hours and 15 minutes, and there was a blood loss during surgery of approximately 160 ml. Postoperative

analgesia was achieved immediately using intravenous analgesics and later (on the 1^st^ and 2^nd^ postoperative days) by using paracetamol.

As a postoperative complication, the patient developed right-side pneumothorax, which was completely drained away in the immediate postoperative period. The drain remained in place for 48 hours and the patient was released on the 4^th^ day after surgery (a total length of hospitalization of four days), with no restrictions.

The patient returned to normal activities 3 weeks after the surgery.

## DISCUSSION

Ablative laparoscopy has become widely used in urology over recent times, and in some cases, it has become the preferred access in patients with various urological pathologies requiring surgery, especially simple nephrectomy, radical nephrectomy, nephroure- terectomy and adrenalectomy.^1^

With regard to adult cystic disease, the role of ablative laparoscopy is mainly related to renal decortication surgery for the relief of pain and symptoms related to large kidney volumes (dyspepsia, respiratory restrictions).^3^ It yields one-year symptom remission rates that vary from 80 to 92%.^1^

Nephrectomy is indicated in cases of hard-to-control hypertension, recurring pyelonephritis and substantial protein loss in the urine. The classical access via bilateral lumbotomy or median incision^2,3^ brings with it the complications that are associated with large-amplitude surgical incisions, with a high morbidity rate of between 12 and 58.7%.^1,2^

Elashy et al. described the two first cases of laparoscopic nephrectomy in patients with adult cystic kidney disease in its terminal stage, with a length of surgery of 273 minutes for the extraction and lengths of hospitalization of 2 and 3 days.^1^

Hand-assisted laparoscopic access has being used in selected cases, when an additional incision is necessary, so as to remove the kidney intact (live donor nephrectomy) and in cases of large kidneys. Comparative studies of laparoscopic nephrectomy versus hand-assisted laparoscopic nephrectomy have proven that the hand-assisted technique is faster, because the use of the hand improves the presentation of the structures, thereby maintaining the advantage of the minimally invasive surgery in relation to surgical recovery and post-surgical pain control.^4^

Schmidlin and Iselin described the first hand-assisted bilateral nephrectomy in a patient with polycystic disease in its final stage, with a length of surgery of 3 hours and 30 minutes. They described this hand-assisted access as fast and safe.^2^

Gill et al. observed the superiority of the laparoscopic method in comparison with open surgery, when performed via the retroperitoneum. If we compare open surgery with retroperitoneoscopy, we observe significant advantages with regard to the length of hospitalization (1.5 versus 9 days), and a faster return to normal activities, with a length of surgery of 4.4 hours. As disadvantages of this method we may include the need to reposition the patient for access to the kidney on the opposite side, and the incision for the removal of the kidney.^3^ In this case, we observed that one advantage of the procedure performed via transperitoneal access was the possibility of access to the two kidney units, with no need for repositioning the patient.

With regard to access to the renal hilum, the supra-umbilical incision was appropriate for safe dissection of the renal hilum and ligature of the vessels.

We believe that the assistance of the hand, in addition to reducing the length of surgery because of the effective presentation, makes the procedure safer because the possibility of touch during surgery results in more comfort for the surgeon.^3^

As an alternative for the access to the renal vessels, one port can be added at each side, and the approach to the hilum can be made under laparoscopic view.

The removal of the kidney was, in this case, done via the supra-umbilical incision after sectioning some cysts and performing external aspiration, thereby preventing the contamination of the abdominal cavity with the content of the cysts.

We believe that the large kidney volume and presence of pneumoperitoneum may partly explain the presence of pneumothorax as a complication. This complication, however, is of no great clinical consequence, and does not affect the length of the patient's hospitalization.

## CONCLUSION

Bilateral laparoscopic nephrectomy can be performed in patients with cystic kidney disease can, according to reports in the literature, be performed either via an exclusively laparoscopic route, with an incision for the removal of the kidney, or via a hand-assisted technique. Both of these offer the patient the advantages of minimally invasive surgery, with minimum pain in the postoperative period and a quick return to daily activities.

We also believe that the hand-assisted technique is feasible, safe, because of the assistance of the hand, and provides for shorter lengths of surgery because of the effective presentation offered by the hand inside the surgical area.

A study with more cases is necessary to prove the real value of this surgical technique in patients with adult polycystic kidney disease.
